# Persistent Lymph Node Metastases After Neoadjuvant Chemoradiotherapy for Rectal Cancer

**DOI:** 10.1001/jamanetworkopen.2024.32927

**Published:** 2024-09-12

**Authors:** Markus Diefenhardt, Daniel Martin, Ralf-Dieter Hofheinz, Michael Ghadimi, Emmanouil Fokas, Claus Rödel, Maximilian Fleischmann

**Affiliations:** 1 Goethe-University Frankfurt, University Hospital, Department of Radiotherapy, Frankfurt, Germany; 2 University Heidelberg, University Hospital, Department of Medical Oncology, Heidelberg, Germany; 3 University Göttingen, University Hospital, Department of General, Visceral and Pediatric Surgery, Göttingen Germany; 4 German Cancer Research Center (DKFZ), Heidelberg, German Cancer Consortium (DKTK), Partner Site Frankfurt am Main, Frankfurt, Germany; 5 Frankfurt Cancer Institute, Frankfurt, Germany; 6 Faculty of Medicine and University Hospital Cologne, Department of Radiation Oncology, Cyberknife and Radiation Therapy, University of Cologne, Cologne, Germany

## Abstract

**Importance:**

Patients with locally advanced rectal cancer and persistent lymph node metastases (PLNM) after neoadjuvant treatment are at high risk of developing locoregional and distant metastasis, yet optimal postsurgical treatment of these patients is limited.

**Objective:**

To analyze the association of PLNM with pretreatment clinical parameters, intensity of neoadjuvant treatment, and long-term oncological outcomes.

**Design, Setting, and Participants:**

This cohort study is a post-hoc analysis of 3 randomized clinical trials (Surgical Oncology Working Group of Germany [CAO], Radiological Oncology Working Group of Germany [ARO], and Working Group for Internal Oncology in the German Cancer Society [AIO]) conducted in Germany in 1994, 2004, and 2012 that included 1948 patients with locally advanced rectal cancer recruited between February 1995 and January 2018. Statistical analysis was conducted between September 2023 and February 2024.

**Exposures:**

Receiving preoperative fluorouracil-based chemoradiotherapy (CRT, comprising the preoperative group of CAO/ARO/AIO-94 and the control group of CAO/ARO/AIO-04), fluorouracil-based CRT plus oxaliplatin (experimental group of CAO/ARO/AIO-04), or total neoadjuvant treatment (TNT) with fluorouracil-based CRT plus oxaliplatin with induction or consolidation leucovorin calcium (folinic acid), fluorouracil, and oxaliplatin chemotherapy within the CAO/ARO/AIO-12 trial.

**Main Outcome and Measures:**

The associations of PLNM with clinical parameters, intensity of neoadjuvant treatment, and cumulative incidences of LR, DM, and overall survival were assessed.

**Results:**

A total of 1888 patients (1333 male participants [70.6%]; median [range] age, 62 [19-84] years) with locally advanced rectal adenocarcinoma (clinical tumor stage 3 to 4 and/or clinically node-positive) treated within 3 consecutive clinical trials were analyzed. A total of 522 (29%) experienced PLNM; 378 had lymph node stage (ypN) 1 (20%) after neoadjuvant treatment (ypN) 1 (20%), and 174 had ypN2 (9%). Age, clinical T-stage, N-stage, grading, carcinoembryonic antigen levels, and time interval from completion of CRT to surgery were significantly associated with PLNM, whereas sex and tumor location were not. The percentage of patients with ypN2 stage was almost halved after TNT (18 of 293 patients [6%]) compared with patients treated with fluorouracil-based CRT (114 of 1009 patients [11.3%]; χ^2^_6_ = 16.693; *P* = .01). After a median (IQR) follow-up of 54 (37-62) months, 5-year overall survival was 86.1% (95% CI, 83.9%-88.4%) for ypN0, 74.0% (95% CI, 83.9%-88.4%) for ypN1, and 43% for ypN2 (95% CI, 35.4%-52.2%) (*P* < .001). The 5-year cumulative incidences of locoregional and distant metastases were, respectively, 3% (95% CI, 2.1%-4.2%) and 20% (95% CI, 18%-23%) for ypN0, 6% (95% CI, 3.4%-8.8%) and 40% (95% CI, 34%-46%) for ypN1, and 19% (95% CI, 13%-26%) and 72% (95% CI, 63%-79%) for ypN2 (both *P* < .001).

**Conclusions and Relevance:**

In this cohort study, PLNM unmasked an unfavorable phenotype of rectal cancer at high risk for treatment failure. More aggressive adjuvant treatment might be considered; however, risk-adapted surveillance strategies and early recurrence-directed surgery, if feasible, are important strategies in this group of patients with CRT- and/or chemotherapy-resistant disease.

## Key Points

**Question:**

To what extent is the persistence of lymph node metastases after neoadjuvant treatment for rectal cancer associated with long-term oncological outcome?

**Findings:**

In this cohort study, 1888 patients treated in 3 consecutive trials of the German Rectal Cancer Study Group were included. Persistent lymph node metastases (especially lymph node stage 2 after neoadjuvant treatment) were associated with younger age, a higher incidence of locoregional recurrence and distant metastases, and worse survival compared with patients without lymph node metastases.

**Meaning:**

These findings suggest intensive surveillance protocols need to be implemented to allow early detection of progression and to enable recurrence-directed strategies.

## Teaser

This cohort study evaluates the association of persistent lymph node metastases with pretreatment clinical parameters, intensity of neoadjuvant treatment, and long-term oncological outcomes using data from 3 randomized clinical trials.

## Introduction

Patients with persistent lymph node metastases (PLNM) after neoadjuvant treatment and surgery for locally advanced rectal cancer are at high risk of developing locoregional (LR) and distant recurrences (DM).^1^ PLNM reflect both the more aggressive biology of malignant cells that have spread to regional lymph nodes and their resistance toward radiotherapy and chemotherapy.

By analyzing our phase 3 trial of preoperative vs postoperative fluorouracil-based chemoradiotherapy (CRT) in patients with locally advanced rectal cancer, we previously demonstrated that preoperative fluorouracil-based CRT sterilized almost half of the lymph-node metastases (patients with pathologically confirmed lymph node metastasis in at least 1 lymph node after neoadjuvant treatment [ypN+]: 25% vs patients with pathologically confirmed lymph node metastasis without neoadjuvant treatment [pN+]: 40% after primary surgery), yet 10-year disease-free survival was 10% worse for the group of PLNM as compared with patients who were pN+ and received postoperative fluorouracil-based CRT, indicating that down-stage migration induced by fluorouracil-based CRT unmasks a subgroup of patients with PLNM with a particularly dismal prognosis.^2^

Meanwhile more intensive neoadjuvant treatment approaches, including multidrug-combination CRT and total neoadjuvant therapy (TNT) have emerged.^3,4^ We here analyze the association of PLNM with pretreatment clinicopathologic parameters and intensity of neoadjuvant treatment on long-term oncologic outcomes in patients treated within 3 consecutive randomized trials of the German Rectal Cancer Study Group (GRCSG). The treatments used in the trial were neoadjuvant fluorouracil-based CRT, neoadjuvant fluorouracil-based CRT plus oxaliplatin, and TNT with fluorouracil-based plus oxaliplatin with 3 cycles of induction or consolidation leucovorin calcium (folinic acid), fluorouracil, and oxaliplatin (FOLFOX) before surgery.^5,6,7,8,9,10^

## Methods

This cohort study included patients with cT3 to 4 and/or clinically node-positive rectal cancer recruited between February 1995 and January 2018 in the CAO/ARO/AIO-94, CAO/ARO/AIO-04, and CAO/ARO/AIO-12 trials of the GRCSG. The design and oncological results have been previously published.^5,6,7,8,9,10^ All sites obtained ethical committee approval and written informed consent from patients. No information on race and/or ethnicity were available. This report follows the Strengthening the Reporting of Observational Studies in Epidemiology (STROBE) reporting guidelines for cohort studies.^14^

### Statistical Analysis

Associations between ypN-stage, pretreatment clinicopathologic factors, time to surgery, and intensity of treatment were analyzed using χ^2^ tests or Kruskal-Wallis tests with Dunn-Bonferroni post hoc tests. OS and cumulative incidence of LR or DM after curative resection (resection status [R] 0 or 1 and distant metastases [M] 0) were plotted using Kaplan-Meier. If the proportional hazards assumption was not fulfilled, restricted mean survival time or accelerated failure time (AFT) models were used to compare long-term outcomes. AFT parametric models provide an alternative to proportional hazard models even considering their limitation in flexibility of distribution chosen.^11^ In brief, an AFT model assumes that a covariate accelerates or decelerates the time to event. A positive regression coefficient means the covariate accelerates the time to event, therefore lengthening survival times. A negative coefficient means the covariate decelerates the time to event, therefore shortening survival time.^12,13^ If the proportional hazard assumption was fulfilled, a competing risk regression model with death as the competing risk was used to compare incidences. All analyses were performed using SPSS version 29 (IBM) and R version 4.3.2 (R Project for Statistical Computing). A *P* value less than .05 was considered significant, and all statistical tests were 2-sided if feasible. Statistical analysis was conducted between September 2023 and February 2024.

## Results

Of the 1948 patients treated in the 3 trials, 60 were excluded due to incomplete data. Of the 1888 eligible patients (1333 male participants [70.6%]; median [range] age, 62 [19-84] years) 1009 had received preoperative fluorouracil-based CRT, 586 had received fluorouracil-based CRT plus oxaliplatin, and 293 had received TNT with fluorouracil-based CRT plus oxaliplatin with induction or consolidation FOLFOX. PLNM were detected in 552 patients (29%); 378 had ypN1 (20%), and 174 had ypN2 (9%) (Table 1). The median (IQR) number of resected lymph nodes was 15 (10-20) in the CAO/ARO/AIO-94 trial, 15 (12-21) in the CAO/ARO/AIO-04 trial, and 16 (13-20) in the CAO/ARO/AIO-12 trial (data available in 1872 patients) (test statistic, 12.708_2_; *P* = .002; CAO/ARO/AIO-94 vs CAO/ARO/AIO-04 test statistic, −103.301; SE, 31.483; standardized test statistic, −3.281; adjusted *P* = .003; CAO/ARO/AIO-94 vs CAO/ARO/AIO-12 test statistic, −126.716; SE, 42.255; standardized test statistic, −2.999; adjusted *P* = .01; CAO/ARO/AIO-04 vs CAO/ARO/AIO-12 test statistic, −23.416; SE, 35.763; standardized test statistic, −0.655; adjusted *P* > .99). Patients with ypN2 were significantly younger compared with patients with ypN1 and ypN0. The median (IQR) age was 62 (55-69) years in patients with ypN0, 62 (55-69) years in patients with ypN1, and 59 years (54-67) years in patients with ypN2 (test statistic, 7.327_2_; *P* = .03; ypN0 vs ypN2 adjusted test statistic, 118.439; SE, 43.937; standardized test statistic, 2.696; *P* = .02). Pretreatment T-stage, N-stage, grading, and carcinoembryonic antigen (CEA) levels were also significantly associated with PLNM, whereas sex and tumor location were not. Median (IQR) CEA levels in patients with ypN0 were 2.8 (1.5-6.3) ng/ml compared with 4.1 (1.8-8.7) ng/ml in patients with ypN1 and 5.0 (2.2-16.5) ng/ml in patients with ypN2 (test statistic, 27.764_2_; *P* < .001; ypN0 vs ypN1 test statistic, −178.651; SE, 39.994; standardized test statistic, −4.467; adjusted *P* = .002; ypN0 vs ypN2 test statistic, −90.954; SE, 26.393; standardized test statistic, −3.446; adjusted *P* < .001). The percentage of patients with ypN2 stage was almost halved after TNT (18 of 293 patients [6%]) compared with patients treated with fluorouracil-based CRT (114 of 1009 patients [11.3%]) and reduced compared with patients treated with fluorouracil-based CRT plus oxaliplatin (42 of 586 patients [7.2%]) (χ^2^ = 16.693_6_; *P* = .01). Time interval from completion of CRT to surgery was significantly longer in patients with ypN0 than in patients with ypN2 (mean [SD], 42 [22] days vs 40 [16] days; test statistic, 137.335; SE, 43.845; standardized test statistic, 3.132; adjusted *P* = .01).

**Table 1.** Baseline Clinical Characteristics by Lymph Node Stage After Neoadjuvant Treatment (ypN) Stage

Parameter	Participants, No./total No. (%)			*P* value^a^
	ypN0	ypN1	ypN2	
Age, y				
19.1-55.0	333/488 (68.2)	103/488 (21.1)	52/488 (10.7)	.43
55.1-62.4	317/457 (69.4)	92/457 (20.1)	48/457 (10.5)	
62.5-68.9	340/471 (72.2)	91/471 (19.3)	40/471 (8.5%)	
69.0-84.4	346/472 (73.3)	92/472 (19.5)	34/472 (7.2%)	
Sex				
Male	935/1333 (71.5)	273/1333 (20.5)	126/1333 (9.5)	.59
Female	402/555 (72.4)	105/555 (18.9)	48/555 (8.6)	
cT stage				
1-2	254/320 (79.4)	46/320 (14.4)	20/320 (6.3)	.002
3-4	1029/1483 (69.4)	317/1483 (21.4)	137/1483 (9.2)	
cN stage				
cN0	368/485 (75.9)	87/485 (17.9)	30/485 (6.2)	.01
cN positive	936/1351 (69.3)	278/1351 (20.6)	137/1351 (10.1)	
Localization, cm				
0-5	527/746 (70.6)	146/746 (19.6)	72/746 (9.7)	.93
>5-10	664/932 (71.2)	188/932 (20.2)	80/932 (8.6)	
>10	124/179 (69.3)	37/179 (20.7)	18/179 (10.1)	
Grading				
1	59/83 (71.1)	21/83 (25.3)	3/83 (3.6)	<.001
2	1078/1481 (72.8)	288/1481 (19.4)	115/1481 (7.8)	
3	80/168 (47.6)	42/168 (25)	46/168 (27.4)	
Treatment				
Fluorouracil-based CRT	695/1009 (68.9)	200/1009 (19.8)	114/1009 (11.3)	
Fluorouracil-based CRT plus oxaliplatin	414/586 (70.6)	130/586 (22.2)	42/586 (7.2)	.01
CRT plus CT	114/144 (79.2)	21/144 (14.6)	9/144 (6.3)	
CT plus CRT	113/149 (75.8)	27/149 (18.1)	9/149 (6.0)	
CEA levels, ng/mL^b^				
≤3	528/687 (76.9)	118/687 (17.2)	41/687 (6.0)	<.001
>3	441/684 (64.5)	175/684 (25.6)	68/684 (9.9)	
Hemoglobin levels, g/dL^b^				
≤13.9	540/774 (69.8)	171/774 (22.1)	63/774 (8.1)	.45
>13.9	523/721 (72.5)	141/721 (19.6)	57/721 (7.9)	
Leucocyte counts, No./nL^b^				
≤7.4	535/754 (71.0)	167/754 (22.1)	52/754 (6.9)	.17
>7.4	526/739 (71.2)	145/739 (19.6)	68/739 (9.2)	
Neutrophil counts, No./nL^b^				
≤4.86	430/600 (71.7)	131/600 (21.8)	39/600 (6.5)	.18
>4.86	425/598 (71.1)	118/598 (19.7)	55/598 (9.2)	
Thrombocyte counts, No./nL^b^				
≤270	526/747 (70.4)	161/747 (21.6)	60/747 (8.0)	.81
>270	536/737 (72.7)	151/737 (20.5)	50/737 (6.8)	
Protein levels, g/dL^b^				
≤7.2	522/725 (72.0)	143/725 (19.7)	60/725 (8.3)	.23
>7.2	427/606 (70.5)	139/606 (22.9)	40/606 (6.6)	
Albumin levels, g/dL^b^				
≤4.2	407/559 (72.8)	115/559 (20.6)	37/559 (6.6)	.10
>4.2	343/507 (67.7)	115/507 (22.7)	49/507 (9.7)	

Abbreviations: CEA, carcinoembryonic antigen; CRT, chemoradiotherapy; CT, chemotherapy; TNT, total neoadjuvant treatment.

SI conversion factors: To convert albumin to grams per liter, multiply by 10.0; CEA to micrograms per liter, multiply by 1.0; hemoglobin to grams per liter, multiply by 10; protein to grams per liter, multiply by 10.0.

^a^ Calculated using χ^2^ tests.

^b^ The median was used to dichotomize the cohort.

The median (IQR) follow-up for the entire cohort after curative resection (R0 or 1, M0; 1787 patients) was 54 (37-62) months. Kaplan-Meier plots for OS and cumulative incidence of DM and LR are shown in Figure. OS, cumulative incidence of LR, and DM were associated with the ypN stage: the 5-year OS in patients with ypN1 was 74.0% (95% CI, 83.9%-88.4%; mean OS, 54; 95% CI, 52-55 months; *P* = .01]), and it was 43.0% (95% CI, 35.4%-52.2%; mean OS, 45 [42-48] months; *P* < .001]) in patients with ypN2 compared with 86.1% (95% CI, 83.9%-88.4%; mean OS, 56 [55-56] months) in patients with ypN0. The 5-year cumulative incidence of LR was 3% (95% CI, 2.1%-4.2%) for ypN0, 6% (95% CI, 3.4%-8.8%) for ypN1, and 19% (95% CI, 13%-26%) for ypN2. The 5-year cumulative incidence of DM was 20% (95% CI, 18%-23%) for ypN0, 40% (95% CI, 34%-46%) for ypN1, and 72% (95% CI, 63%-79%) for ypN2. In a regression model with death as a competing risk, the risk of distant metastasis (ypN0 reference; ypN1 hazard ratio [HR], 2.82; 95% CI, 2.26-3.52; ypN2 HR, 6.74; 95% CI, 5.28-8.59) was significantly associated with ypN stage (both *P* < .001). The median (IQR) time to distant metastasis in patients with ypN2 was only 11 (5-21) months. In a regression model adjusted for treatment, ypN2 stage was significantly associated with OS, LR, and DM, but neither treatment approach nor the interaction term between treatment approaches and ypN stage were significantly associated with long-term oncological outcome (Table 2).

**Figure.**
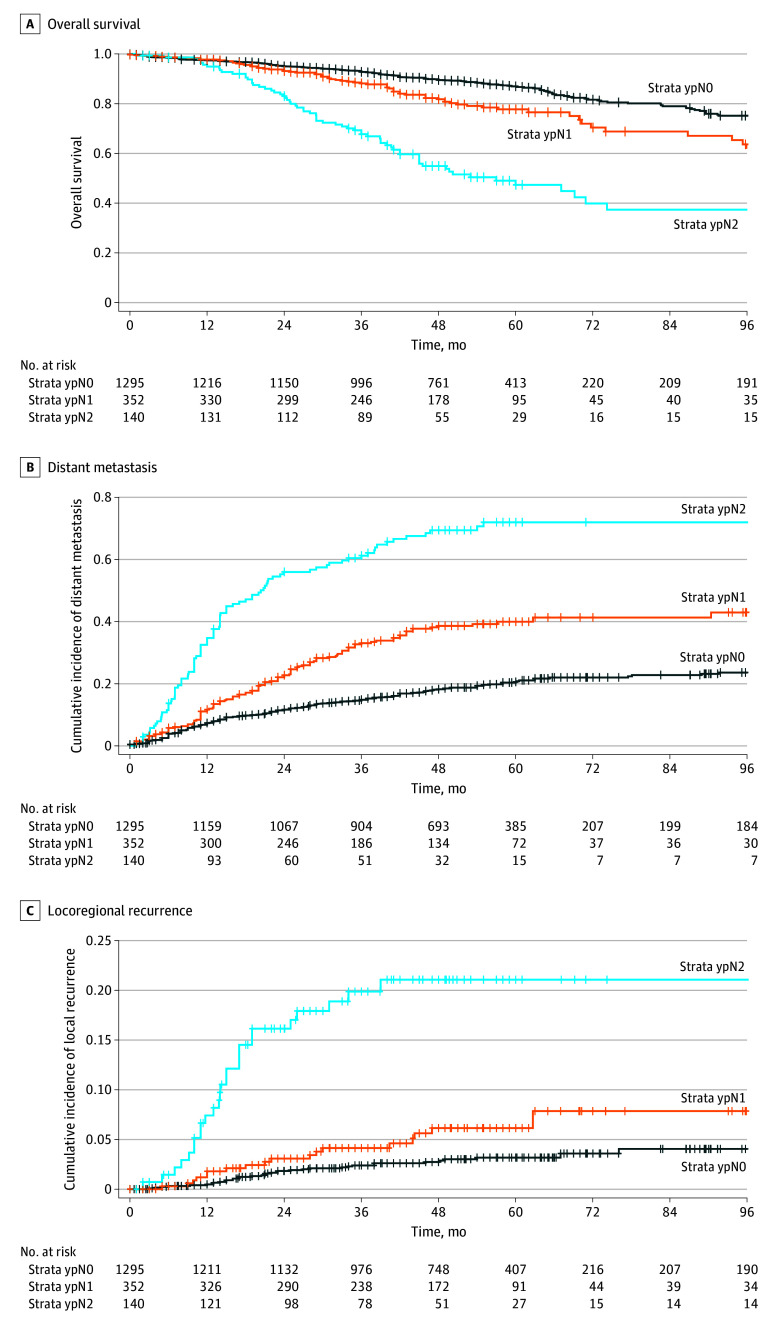
Overall Survival, Incidence of Distant Metastasis, and Incidence of Locoregional Recurrence Stratified by Pathological Lymph Node Stage After Neoadjuvant Treatment (ypN) Five-year overall survival, mean overall survival after 5-years, incidences of locoregional recurrence and distant metastasis are reported.

**Table 2.** Overall Survival, Incidence of Locoregional Recurrence and Distant Metastasis by Lymph Node Stage After Neoadjuvant Treatment (ypN) Stage^a^

Accelerated failure time model distribution	OS		LR		DM	
	Lognormal coefficient (95% CI)	*P* value	Lognormal coefficient (95% CI)	*P* value	Lognormal coefficient (95% CI)	*P* value
ypN Stage						
0	1 [Reference]	NA	1 [Reference]	NA	1 [Reference]	NA
1	−0.43 (−0.74 to −0.12)	.01	−0.51 (−1.38 to 0.35)	.24	−1.25 (−1.73 to −0.76)	<.001
2	−1.24 (−1.60 to −0.87)	<.001	−2.54 (−3.42 to −1.67)	<.001	−2.90 (−3.47 to −2.31)	<.001
Treatment						
Fluorouracil-based CRT	1 [Reference]	NA	1 [Reference]	NA	1 [Reference]	NA
Fluorouracil-based CRT plus oxaliplatin	0.02 (−0.26 to 0.30)	.89	0.58 (−0.35 to 1.50)	.22	0.11 (−0.34 to 0.56)	.63
TNT	0.28 (−0.12 to 0.68)	.17	−0.51 (−1.32 to 0.31)	.22	−0.27 (−0.78 to 0.24)	.30
Interaction terms						
factor(Arm)Fluorouracil-based CRT plus oxaliplatin × factor(ypN1)	0.12 (−0.42 to 0.66)	.67	−0.66 (−2.18 to 0.87)	.40	−0.19 (−0.97 to 0.60)	.65
factor(Arm)TNT × factor(ypN1)	−0.33 (−1.09 to 0.42)	.38	−0.40 (−2.02 to 1.23)	.63	−0.10 (−1.11 to 0.91)	.85
factor(Arm)Fluorouracil-based CRT plus oxaliplatin × factor(ypN2)	−0.11 (−0.83 to 0.60)	.76	−0.71 (−2.36 to 0.94)	.40	0.18 (−0.91 to 1.27)	.75
factor(Arm)TNT × factor(ypN2)	0.40 (−0.63 to 1.43)	.45	0.92 (−1.11 to 2.96)	.37	1.32 (−0.13 to 2.76)	.07

Abbreviations: CRT, chemoradiotherapy; DM, incidence of distant metastasis; LR, incidence of locoregional recurrence; NA, not applicable; OS, overall survival; TNT, total neoadjuvant treatment.

^a^ A multivariate accelerated failure time model was used as alternative to a Cox regression model as the proportional hazard assumption was violated. Positive coefficients imply the time to event is lengthened; hence, the hazard rate is decreasing. Negative coefficients imply the time to event is shortened; hence, the hazard rate is increasing. Lognormal distribution was selected after visual inspection.

## Discussion

We confirm that patients with PLNM carry a high risk of early treatment failure. PLNM were significantly associated with pretreatment T/N-stage, grading, and CEA levels. We also identified younger age as a significant factor, in line with a previous analysis from Calvo et al^15^ in 487 patients with rectal cancer that had received various forms of neoadjuvant treatment and surgical resection over a period of 20 years. Of note, using the Surveillance, Epidemiology, and End Results database, younger age was also associated with an increased risk of positive lymph node metastases in patients with rectal cancer after primary surgery.^16^

Intensification of neoadjuvant treatment and/or extending the interval from completion of CRT to surgery can reduce PLNM. In the recent Rectal Cancer and Preoperative Induction Therapy Followed by Dedicated Operation (RAPIDO) and PRODIGE 23 trials, TNT reduced the incidence of ypN2 compared with fluorouracil-based CRT (7% vs 12% and 3% vs 9%, respectively), and in both trials this was associated with superior long-term outcomes.^3,4,17^ In the RAPIDO trial, in line with the American Society for Radiation Oncology recommendation, a boost after 45 Gy was only recommended to the tumor bed, not to the lymph nodes.^18,19^ Notwithstanding the limitations of retrospective comparison, our pooled analyses confirm that more intense neoadjuvant treatment may lead to a reduction of PLNM, yet patients with ypN2 are still at high risk of treatment failure irrespective of preoperative treatment intensity.

Given that the cumulative incidence of DM after curative resection reached 40% for ypN1 and 72% for ypN2 at 5 years, more aggressive adjuvant treatment might be considered for these subgroups. However, data from randomized trials do not support a clinical benefit of this strategy. In a meta-analysis of individual patient data from 4 randomized trials comparing observation with adjuvant chemotherapy after preoperative (chemo)radiotherapy, Breugom et al^20^ did not show any significant benefit of adjuvant chemotherapy for OS, disease-free survival, or DM. Indeed, the subgroups of patients with ypN1 and especially ypN2 seemed to benefit least from adjuvant chemotherapy.

In such a scenario, we recommend intensification of surveillance protocols for PLNM, especially in the first 2 years to identify treatment failure early on and possibly enable recurrence-directed surgery. Data from the RAPIDO trial support this strategy as the median time from randomization to DM was only 1.4 and 1.3 years after TNT and CRT, respectively, and 73% and 78% of DM, respectively, were diagnosed in 1 organ site only.^21,22^ Indeed, 46% of patients treated with TNT and 52% of patients treated with CRT in the RAPIDO trial underwent salvage surgery for DM. Of note, their analysis did not discriminate between ypN stages, and it remains unclear whether ypN1 or ypN2 may correlate with earlier multiorgan, rather than single site progress and, thus, undermine curative local treatment.

### Limitations

Our study has several limitations. First, this is a post hoc secondary analysis of 3 independent studies over a period of more than 20 years. During this period, in addition to improved salvage treatment options that may affect overall survival, modern radiation delivery techniques and surgical developments, with an increase in the number of lymph nodes resected in recent trials, should be considered when interpreting our results.^23^ Second, the inclusion criteria of the 3 studies differed slightly, with magnetic resonance imaging only being mandatory in CAO/ARO/AIO-12. Third, the treatments evolved, especially with respect to (neo-)adjuvant chemotherapy combinations and cycles. Fourth, this cohort study may be underpowered to identify a significant effect of neoadjuvant treatment strategies to the association between ypN stage and long-term outcome.

## Conclusions

In this cohort study, we showed that PLNM unmask an aggressive phenotype of rectal cancer at high-risk for treatment failure irrespective of intensity of neoadjuvant treatment. Early initiation of recurrence-directed surgery, if feasible, is an important strategy in this group of patients with CRT and/or chemotherapy resistant disease.

## Notes

Supplement.
Data Sharing Statement
