# Supplementary material for: Persistent Lymph Node Metastases After Neoadjuvant Chemoradiotherapy for Rectal Cancer
Source: JAMA Netw Open. 2024 Sep 12;7(9):e2432927. doi: 10.1001/jamanetworkopen.2024.32927 (PMC11393720; doi:10.1001/jamanetworkopen.2024.32927)
Supplement: Supplement. — Data Sharing Statement [file jamanetwopen-e2432927-s001.pdf]

Data Sharing Statement

Diefenhardt. Persistent Lymph Node Metastases After Neoadjuvant Chemoradiotherapy for With Rectal Cancer. *JAMA Netw Open*. Published September 12, 2024. doi:10.1001/jamanetworkopen.2024.32927

Data
Data available: No

Additional Information
Explanation for why data not available: Patients enrolled in the trials did not provide informed consent for the data to be shared publicly. Therefore, data from the trials cannot be shared publicly.
